# PEEP titration: the effect of prone position and abdominal pressure in an ARDS model

**DOI:** 10.1186/s40635-018-0170-9

**Published:** 2018-01-30

**Authors:** Joseph C. Keenan, Gustavo A. Cortes-Puentes, Lei Zhang, Alex B. Adams, David J. Dries, John J. Marini

**Affiliations:** 1 University of Minnesota, Minneapolis, MN USA; 2 Regions Hospital, Saint Paul, MN USA; 3 Mayo Clinic Department of Pulmonary and Critical Care, Rochester, MN USA; 4 Pulmonary/Critical Care Medicine, Regions Hospital, 640 Jackson Street, St. Paul, MN 55101 USA

**Keywords:** Mechanical ventilation, Intra-abdominal hypertension, Esophageal pressure, Chest wall

## Abstract

**Background:**

Prone position and PEEP can both improve oxygenation and other parameters, but their interaction has not been fully described. Limited data directly compare selection of mechanically “optimal” or “best” PEEP in both supine and prone positions, either with or without changes in chest wall compliance.

To compare best PEEP in these varied conditions, we used an experimental ARDS model to compare the mechanical, gas exchange, and hemodynamic response to PEEP titration in supine and prone position with varied abdominal pressure.

**Methods:**

Twelve adult swine underwent pulmonary saline lavage and injurious ventilation to simulate ARDS. We used a reversible model of intra-abdominal hypertension to alter chest wall compliance. Response to PEEP levels of 20,17,14,11, 8, and 5 cmH_2_O was evaluated under four conditions: supine, high abdominal pressure; prone, high abdominal pressure; supine, low abdominal pressure; and prone, low abdominal pressure. Using lung compliance determined with esophageal pressure, we recorded the “best PEEP” and its corresponding target value. Data were evaluated for relationships among abdominal pressure, PEEP, and position using three-way analysis of variance and a linear mixed model with Tukey adjustment.

**Results:**

Prone position and PEEP independently improved lung compliance (*P* < .0001). There was no interaction. As expected, intra-abdominal hypertension increased the PEEP needed for the best lung compliance (*P* < .0001 supine, *P* = .007 prone). However, best PEEP was not significantly different between prone (12.8 ± 2.4 cmH_2_O) and supine (11.0 ± 4.2 cmH_2_O) positions when targeting lung compliance

**Conclusions:**

Despite complementary mechanisms, prone position and appropriate PEEP exert their positive effects on lung mechanics independently of each other.

## Background

The mechanically heterogeneous lung affected by acute respiratory distress syndrome (ARDS) is difficult to ventilate safely. An appropriate setting for positive end-expiratory pressure (PEEP) helps stabilize recruitment and reduces the number of lung units subjected to stress focusing and tidal opening and closure. The benefits of PEEP, however, are accompanied by risks of increased global parenchymal strain, raised pulmonary vascular resistance, and impeded venous return. These competing effects underline the importance of achieving an appropriate balance in the individualized application of PEEP.

The physiologic effects of PEEP depend on pulmonary and extra-pulmonary factors that are not accounted for in commonly used PEEP:FIO_2_ tables. Arterial oxygenation may reflect lung recruitment but is influenced by other factors, including venous oxygen tension and the distributions of lung perfusion and ventilation. Alternative parameters for titrating PEEP include measures of thoracic mechanics, ventilatory dead space [1], and other gas exchanging and hemodynamic targets [2, 3]. Currently, there is no consensus regarding the best technique for adjusting PEEP. Consequently, its use in trials and clinical practice varies widely.

As first elaborated in Suter’s classic study of “best PEEP” [4], tidal thoracic mechanics offers a viable option for PEEP titration, correlating well with oxygen delivery (as opposed to oxygen tension) and ventilatory dead space fraction. The tidal compliance (directly) and the driving pressure (inversely) associated with a given tidal volume may reflect the balance between overdistention and recruitment of functional units. Better compliance and reduced driving pressure may positively influence the outcome of ventilation for ARDS [5]. Despite these benefits, titrating PEEP to best respiratory system compliance is controversial.

Prone positioning, an intervention used in conjunction with PEEP for safely ventilating ARDS patients, can improve arterial oxygenation as it alters chest wall and lung compliances and redistributes ventilation. Although PEEP and prone positioning may be additive in improving oxygenation [6], the *mechanical* response of the lung to PEEP in the prone position, which is theoretically important to lung protection and ventilator-induced lung injury (VILI) avoidance, has not been extensively explored or precisely described. Prone positioning might affect not only the PEEP value needed for the same degree of lung opening but also the PEEP level associated with other targets for optimization.

Limited data are available that directly compare selection of mechanically “optimal” or “best” PEEP in both supine and prone positions, either with or without further stiffening of the chest wall by increased intra-abdominal pressure (IAP). To test the hypothesis that prone positioning should alter the level of mechanically “best” PEEP, we conducted an experimental study in pigs using a model of ARDS that focused on evaluating positional lung mechanics and gas exchange. We applied a range of PEEP values in the supine and prone positions, with and without alteration of chest wall compliance by intra-abdominal hypertension (IAH). In the spirit of Suter et al., but with understanding the effects of our intervention on the lung itself, rather than the respiratory system, we defined best PEEP as the PEEP resulting in the best lung compliance.

## Methods

This protocol was approved by the Animal Care and Use Committee of Regions Hospital (St. Paul, MN).

### Animal preparation

Young healthy Yorkshire pigs (*n* = 12, mean weight = 48.1 ± 4.2 kg) were prepared, mechanically ventilated using the Engström Carestation (GE Healthcare, Madison, WI), and saline lavaged in a manner previously described [7]. For manipulation of IAP, the peritoneal cavity was accessed by surgical placement of a gas-tight tracheostomy tube (Covidien Shiley Trach tube 7, Mansfield, MA). A continuous positive airway pressure circuit was connected to the abdominal tracheostomy tube and set to either atmospheric pressure or 20 cmH_2_O (14.7 mmHg) for the normal IAP and IAH phases of the protocol, respectively. The tip of an esophageal balloon catheter was advanced to a depth of approximately 40 to 50 cm from the incisors, the balloon was inflated with 5 ml of air, and 3.5 ml was withdrawn to leave 1.5 ml. Gastric positioning was confirmed by transient increase in pressure during compression of the abdomen and by gastric content return. The esophageal balloon catheter was then withdrawn to a depth of approximately 30 to 40 cm where obvious cardiac oscillations were observed in the tracing.

After saline lavage, the swine were ventilated for 60 min in volume-controlled mode using “square” wave flow, tidal volume (*V*_*T*_) of 15 mL/kg, frequency 15 breaths per minute, inspiratory-expiratory ratio of 1:2, PEEP of 0 cmH_2_O, and fraction of inspired oxygen (FIO_2_) of 100%. Upon completion of these preparatory steps, the ventilator was returned to baseline settings.

### Experimental protocol

We studied each passively ventilated animal subject in both prone and supine positions, at normal IAP and during IAH, for a total of four conditions: supine, normal IAP; supine, IAH; prone, normal IAP; and prone, IAH. The order of conditions tested was randomized.

For each condition, six individual PEEP levels were evaluated (20, 17, 14, 11, 8, and 5 cmH_2_O) in a descending order. This method was intended to mimic the bedside decremental PEEP titration used in clinical practice. Prior to each titration, a recruitment maneuver was performed consisting of pressure control with inspiratory pressure of 10 cmH_2_O above a PEEP of 20 cmH_2_O for 10 breaths. Following this maneuver, the PEEP was set at 20 cmH_2_O and the ventilator was returned to baseline settings. After 5 min, we recorded hemodynamic data and lung mechanics. Arterial blood gases were evaluated 10 min after the PEEP level was established. Functional residual capacity (FRC) was measured using the wash-in/washout method available on the CareStation ventilator. After the collection of data, the PEEP was decreased to 17 cmH_2_O and data were again collected as described above. This was repeated in a similar manner until reaching PEEP 5 cmH_2_O.

When data for all six PEEP levels were collected, the animal was returned to the supine position with intra-abdominal pressure normal (if not already) for 5 min before proceeding to the next randomly selected condition.

### Statistical analysis

Three-way analysis of variance was used to determine the interactions between position, abdominal pressure, and PEEP for each dependent variable: lung compliance (C_LUNG_), chest wall compliance (C_CW_), respiratory system compliance (C_RS_), PAO_2_:FIO_2_ ratio, functional residual capacity (FRC), and cardiac output (CO). Lung compliance was calculated as tidal volume divided by the difference between the end-inspiratory and end-expiratory trans-pulmonary pressure (P_TP_). The best value for each dependent variable was selected, and the PEEP level at each best value was recorded. Linear mixed model was used to compare best value and PEEP level at best value among four conditions in a pairwise fashion: supine position, normal IAP; supine position, IAH; prone position, normal IAP; and prone position, IAH. Tukey method was used to adjust for multiple comparisons. All *P* values are two-sided, and < 0.05 is considered statistically significant. All analyses were carried out using the SAS system (v. 9.3; SAS Institute, Cary, NC, USA).

## Results

### The effects of PEEP and prone position on lung mechanics

#### Lung compliance

**Fig. 1:**
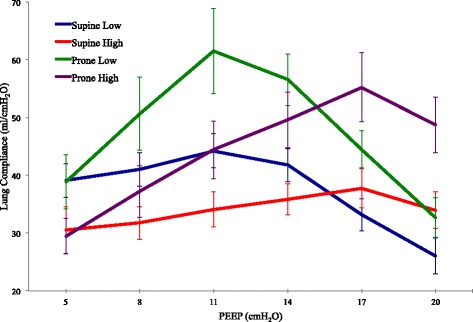
Mean lung compliance for each condition at each PEEP level (±SD)

Prone position and PEEP independently affected C_LUNG_ (*P* < .0001). There was no interaction. This resulted in a best PEEP that did not significantly differ between prone and supine positions when targeting C_LUNG_ (Table 1, Fig. 1), with or without IAH. However, IAH did affect C_LUNG_ response to PEEP. The best PEEP at normal IAP was significantly less than that for IAH, both in prone (*P* = .007) and supine (*P* < .0001) positions.

**Table 1:** Optimum PEEP as determined by a decremental PEEP titration optimizing each of the listed titration targets (A). Optimal titration target value achieved in each condition (B)

Titration target	Supine low	Supine high	Prone low	Prone high
(A)				
C_LUNG_	11.0 (4.2)*^‡^	17.3 (4.3)^†††^	12.8 (2.4)^‡‡^	16.7 (2.1)
P_D_ and C_RS_	10.8 (3.3)*^‡‡‡^	18.8 (2.0)^†^	13.0 (2.3)^‡‡^	16.7 (2.1)
C_CW_	11.0 (3.6)**^‡^	19.0 (2.0)^††^	12.3 (5.0)^‡^	14.3 (3.7)
EEP_TP_	11.0 (2.5)***^‡^	16.5 (2.8)^††^	11.3 (4.3)	14.8 (3.6)
FRC	18.3 (2.4)*	19.5 (1.2)^†††‡‡^	18.3 (1.5)	19.4 (1.2)
PAO_2_:FIO_2_	17.8 (2.3)	17.5 (2.5)	17.8 (2.3)	17.3 (3.4)
CO	6.3 (2.0)***^†‡‡‡^	6.3 (3.0)^†††^	7.0 (2.3)^‡‡^	9.1 (4.5)
(B)				
C_LUNG_ (ml/cmH_2_O)	47.7 (11.3)*^†^	39.6 (12.9)^†^	62.7 (17.8)^‡^	57.2 (21.3)
P_D_ (cmH_2_O)	14.3 (2.9)^*†‡^	17.0 (3.8)	12.6 (2.5)	14.6 (2.8)
C_RS_ (ml/cmH_2_O)	34.6 (6.6)^†^	29.4 (6.3)^†‡^	40.8 (10.1)	34.4 (6.6)
C_CW_ (ml/cmH_2_O)	196.0 (54.2)*^‡^	148.7 (36.3)^†^	145.0 (59.8)	118.1 (33.1)
FRC (ml)	1335 (360)*	693 (161)^†‡^	1277 (374)	970 (256)
PAO_2_:FIO_2_	442 (130)	249 (139)	454 (130)	386.0 (147.2)
CO (L/min)	3.7 (0.7)*	3.7 (0.7)^†‡^	3.5 (0.5)^‡^	3.8 (0.5)

Descriptive statistics are shown as mean ± SD. Supine low represents supine position and normal IAP. Supine high represents supine position and IAH. Prone positions are represented similarly

*C*_*LUNG*_ lung compliance, *P*_*D*_ driving pressure, *C*_*RS*_ respiratory system compliance, *C*_*CW*_ chest wall compliance, *FRC* functional residual capacity, *CO* cardiac output

*p < .05 vs. supine high, **p < .001 vs. supine high, ***p < .0001 vs. supine high, ^†^*p* < .05 vs. prone low, ^††^*p* < .001 vs. prone low, ^†††^*p* < .0001 vs. prone low, ^‡^*p* < .05 vs. prone high, ^‡‡^*p* < .001 vs. prone high, ^‡‡‡^*p* < .0001 vs. prone high

The best C_LUNG_ achieved during decremental PEEP titration was greater for the prone position than for supine. This relative advantage was observed both at normal IAP (*P* = .006) and during IAH (*P* = .003), consistent with the additive relationship between prone position and PEEP.

#### Respiratory system compliance/driving pressure

**Fig. 2:**
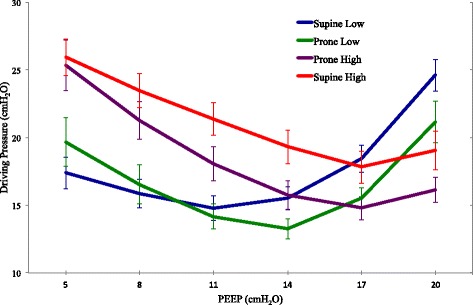
Mean driving pressure for each condition at each PEEP level. The left vertical axis is labeled as driving pressure and the right vertical axis is labeled as respiratory system compliance (±SD)

There was no interaction between prone position and PEEP in their effect on C_RS_. Prone position improved C_RS_ at normal IAP (*P* = .013). IAP influenced the effect of PEEP, with the best PEEP for C_RS_ significantly higher during IAH than at normal IAP (*P* < .0001 supine, *P* = .008 prone) (Table 1, Fig. 2). Because driving pressure has been defined as the quotient of (an unchanging) tidal volume and C_RS_, the same summary statements apply regarding the relative effects of PEEP, prone positioning, and IAP on that indicator of respiratory system mechanics.

#### End-expiratory transpulmonary pressure

There was no interaction between prone position and PEEP in their effect on end-expiratory transpulmonary pressure (EEP_TP_).

**Fig. 3:**
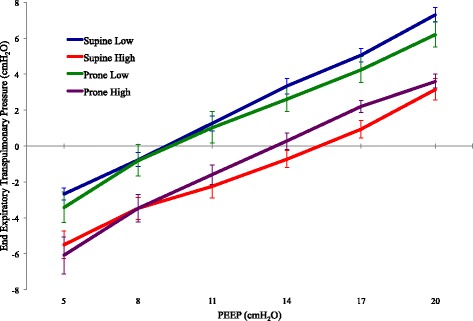
Mean end expiratory transpulmonary pressure for each condition at each PEEP level (±SD)

Prone position resulted in a lower EEP_TP_ than supine position at normal IAP (*P* = 0.028). This effect was not observed during IAH (Table 1, Fig. 3).

At normal IAP, the best PEEP (defined as the lowest PEEP that achieved a positive EEP_TP_) was not different between prone and supine positions. This relationship persisted during IAH.

#### Chest wall compliance

There was no interaction between prone position and PEEP in their effect on C_CW_. Prone positioning decreased C_CW_ at normal IAP (*P* = .004) but had no significant effect during IAH. The effect of PEEP depended on the IAP, with the optimal PEEP for C_CW_ being higher during IAH than at normal IAP (*P* = .027) (Table 1).

### The effects of PEEP and prone position on gas exchange

There was no interaction between PEEP, position, or IAP. Prone positioning improved PaO_2_:FIO_2_ ratio (*P* < .0001). IAH decreased PaO_2_:FIO_2_ ratio (*P* < .0001). There were no differences in best PEEP for PaO_2_:FIO_2_ between conditions (Table 1).

### The effects of PEEP and prone position on cardiac function

#### Cardiac output

PEEP and IAP independently affected cardiac output. CO decreased as PEEP increased. Prone position did not exert an independent effect but did modify the effect of PEEP. At low PEEP (5 cmH_2_O), supine position predicted a higher CO (*P* = .003). Conversely, at a PEEP of 20 cmH_2_O, prone position predicted a higher CO (*P* = .012) (Table 1).

## Discussion

Prone positioning did not consistently alter the mechanically best PEEP value in our ARDS model. Our primary titration target for determining best PEEP was C_LUNG_, but its behavior paralleled the responses of a variety of titration targets, both at normal IAP and during IAH. Moreover, the PEEP that caused transition from negative to positive EEP_TP_ was not altered by proning.

Prone position and PEEP both improve oxygenation [8, 9] and affect homogeneity of ventilation [10, 11] and regional mechanics [12, 13]. Both also may decrease the potential for VILI [14–16]. Reduced mortality was associated with prone positioning of severely ill patients with ARDS in one large and influential clinical trial [17] and with high PEEP in a meta-analysis of studies relating to that intervention [18]. Driving pressure, a variable directly linked to tidal compliance and influenced by both proning and PEEP, appears to correlate with mortality risk in supine patients with ARDS [5].

Despite similar effects on oxygenation and shared capacity to influence driving pressure, supine PEEP and prone positioning distend and re-shape the lung differently. PEEP recruits atelectatic lung units and improves distribution of ventilation via compensatory interdependence through the network lattice. Prone position redistributes ventilation in a more homogeneous manner. Both factors result in improved homogeneity in distribution of ventilation and local P_TP_. The potentially important interaction between prone position and PEEP—whether negative, additive, or synergistic—has not been fully elucidated, especially in the setting of IAH.

The stability of the average best PEEP value between supine and prone positions during our study suggests that values of “lung protective” PEEP determined in the supine position might inappropriately be turned down after proning improvement by clinicians guided by the widely employed PaO_2_:FIO_2_ tables. Our findings suggest that such reductions might not be prudent when VILI prevention is the goal. Because decreasing PEEP lowers the EEP_TP_ (Fig. 3), tidal ventilation would theoretically take place within a lower sector of the lung’s pressure/volume curve, encouraging mechanical heterogeneity and potentially increasing the risk of VILI. Best mechanical PEEP when prone appears, on average, to be similar to the supine value individual animals varied somewhat in their mechanical properties, however; therefore, best PEEP by this criterion did not invariably remain the same after the position change in every animal. Given the steeper contours of the mean C_LUNG_-PEEP curve after pronation, re-titration to a tidal compliance target may be even more advisable for the prone position than for the supine (Fig. 1).

### PEEP titration and choice of “target” variable

Suter and colleagues described an inverted U-shaped curve showing that the best oxygen delivery and tidal compliance occurred at the same PEEP with higher and lower values of PEEP associated with less desirable results. Optimized oxygen delivery is no longer the primary goal of ventilator settings, but the U-shaped PEEP response of the tidal compliance curve remains the inspiration for efforts to individualize PEEP. The heterogeneity of patients presenting with similar syndromes but different disease processes suggests the need for individualized ventilator settings by PEEP titration.

The demonstrated linkage of driving pressure to outcome [5] indicates that C_RS_, the quotient of tidal volume and driving pressure, is a reasonable and clinically accessible variable to be optimized during PEEP titration. Moreover, examination of our PEEP response curves for C_LUNG_ and airway driving pressure (Figs. 1 and 2) suggests that in some (if not most) cases, airway driving pressure during volume controlled ventilation could be substituted for C_LUNG_ if esophageal pressure monitoring is not readily available.

Targeting a positive end-expiratory transpulmonary pressure has been suggested as a useful means by which to adjust PEEP in ARDS [19]. Under the normal IAP conditions of our experiments, esophageal pressure did not change significantly in the transition from supine to prone, despite the changing positions of various thoracic components. Consequently, EEP_TP_ transitioned from negative to positive at a similar PEEP in both the supine and prone positions (Figs. 1 and 3). Accepting the limitations of esophageal pressure estimation of pleural pressure, a PEEP level that keeps EEP_TP_ positive in either position should ensure adequate pressure to keep alveolar units inflated across the horizontal gravitational plane while minimizing the risk of overdistension during the tidal cycle.

### Cardiovascular findings

Both PEEP and position influence cardiac function. The net effect of PEEP on cardiac output is the composite of its effects on the right and left sides of the heart. PEEP decreases cardiac output by limiting right ventricular filling and increasing right heart afterload. By reducing the transmural systolic gradient, PEEP can improve left ventricular function, especially in the setting of decompensated heart failure.

Prone positioning appears to offer several potential mechanisms for improving cardiac output, including decreased afterload and improved venous return [20]. Prone position increased cardiac output in patients found to be volume responsive prior to the maneuver; however, this benefit was absent in patients who were not fluid responsive [20]. The optimal PEEP values for cardiac output in all four position and abdominal pressure groups were lower than the best PEEP for C_LUNG_, but there were no differences in mean values for CO among the four groups. This may have been due to the fact that as a precaution, all animals were volume replete, limiting the likelihood that low intravascular volume would lead to poor tolerance of PEEP during anesthesia.

### The effect of intra-abdominal pressure

IAH stiffens the chest wall, and it reduced C_CW_, C_LUNG_, C_RS_, and EEP_TP_ in a manner similar to that observed in our previous work (Cortes-Puentes). IAP significantly affected PEEP response for each of these variables. As expected, more PEEP was required to reach the optimal value of these variables during abdominal hypertension. Thus, a greater PEEP was required to transition EEP_TP_ from negative to positive, and the curves relating PEEP and EEP_TP_ to each of the compliance variables were shifted to the right (Figs. 1, 2 and 3). Although chest wall compliance during IAH was significantly less when prone than supine, this stiffening was accompanied by improved C_LUNG_, so that C_RS_ remained relatively unchanged by prone positioning during IAH.

We acknowledge that some of our results may be affected by our specific method of raising IAP. It is a controlled pressure model of abdominal hypertension so that prone positioning, while it may decrease overall chest wall compliance, will not increase IAH beyond the pressure set value. Our findings do suggest, however, that the prone positioning may be well tolerated from both respiratory and cardiovascular standpoints in the setting of IAH.

## Conclusions

Viewed with the limitations of a pre-clinical model in mind, these findings suggest conceptual clinical parallels. Logical measures to minimize parenchymal stress would include both prone position and PEEP titration to improve distribution of ventilation, decrease tissue strain, and promote tidal ventilation in the zones of the highest compliance. Our data suggest that whereas increasing IAP raises the PEEP level needed to achieve the best lung compliance, prone repositioning does not consistently influence the PEEP level needed to achieve “best” values for C_LUNG_ and avoid negative end-expiratory transpulmonary pressures determined using an esophageal balloon catheter. Similar best PEEP between positions does not imply equivalence in lung protection. Indeed, the PEEP and prone position independently improved C_LUNG_, and their effects were additive. When esophageal pressure monitoring is not available for estimating C_LUNG_, airway driving pressure appears to be a reasonable surrogate to act as the titration target for mechanically “best” PEEP.

## Abbreviations

ARDS: Acute respiratory distress syndrome
C_CW_: Chest wall compliance
C_LUNG_: Lung compliance
CO: Cardiac output
C_RS_: Respiratory system compliance
EEP_TP_: End-expiratory transpulmonary pressure
FIO_2_: Fraction of inspired oxygen
FRC: Functional residual capacity
IAH: Intra-abdominal hypertension
IAP: Intra-abdominal pressure
PAO_2_:FIO_2_: Ratio of arterial oxygen partial pressure to fraction of inspired oxygen
PEEP: Positive end-expiratory pressure
VILI: Ventilator-induced lung injury
